# A Novelly Developed Bipolar Needle Knife Can Be an Alternative Device Choice for Endoscopic Submucosal Dissection (With Video)

**DOI:** 10.3389/fmed.2022.888635

**Published:** 2022-05-13

**Authors:** Shengsen Chen, Danping Zhou, Jiangping Yu, Rongwei Ruan, Yuanshun Liu, Yandong Li, Qiwen Shen, Shi Wang

**Affiliations:** Department of Endoscopy, Institute of Basic Medicine and Cancer (IBMC), Cancer Hospital of the University of Chinese Academy of Sciences (Zhejiang Cancer Hospital), Chinese Academy of Sciences, Hangzhou, China

**Keywords:** endoscopic submucosal dissection, novel bipolar-current needle-knife, alternative device choice, early digestive tract cancers, monopolar knife

## Abstract

**Background:**

Endoscopic submucosal dissection (ESD) is technically difficult with high rates of complications, such as perforation and bleeding. We aimed to explore the safety and cutting efficiency of a novelly devised bipolar knife for ESD procedure.

**Methods:**

Taking a traditional monopolar knife as a reference, the safety and feasibility of the novel bipolar knife were evaluated by an animal experiment and a human study. Furthermore, we assessed the usefulness and advantage of this novel bipolar knife by using the finite element method.

**Results:**

A porcine experiment confirmed that there was no significant difference in wound size and cutting speed between the monopolar and bipolar knives. The thermal damage and histopathological scores produced by the two knives were similar. In addition, the porcine experiment and patients' study identified that the incidence of postoperative complications, such as bleeding, perforation, and infection, had no statistical difference between the monopolar and bipolar groups. Finally, the finite element model showed that the length and depth of thermal damage caused by the bipolar knife were, respectively, 102.77–117.98% and 80.87–84.53% of those caused by the monopolar knife at the same power.

**Conclusion:**

The novel bipolar knife was theoretically safer than the monopolar knife and, at least, was confirmed not inferior to the monopolar knife in operability and cutting efficiency. Thus, the novel bipolar knife can be an alternative device choice for ESD.

## Introduction

Digestive tract cancers (e.g., esophageal, gastric, and colorectal cancers) are common malignant tumors and major causes of mortality worldwide. The endoscopic submucosal dissection (ESD) is widely used for treating early cancer of digestive tract, which facilitates the en bloc resection of large superficial tumors, reduces the risk of local cancer recurrence, and enables an accurate histopathological diagnosis (1, 2). However, the procedure of ESD for treating early digestive tract cancers, especially the esophagus and colorectum with thinner walls than the stomach, is technically difficult.

Circumferential mucosal incision and dissection of the preinjected submucosal layer are key steps of ESD and are commonly performed by using a monopolar endoscopic electrosurgical knife (1, 2). Nevertheless, the traditional monopolar knife has high requirements for endoscopists' skills and experience. It may also cause perforation, bleeding, and other complications, particularly at the thin anatomic sites of the digestive tract while performing ESD (3, 4). Risk factors for postoperative complications are known as vertical thermal damage to muscularis propria of the digestive tract during operation and poor control of the endoscopic electrosurgical knife (4).

To minimize vertical thermal damage to deeper tissues, Sano et al. (5) designed a bipolar-current needle-knife (B-knife) with a negative electrode attached to the knife on the end of the sheath. In the following year, a ball-tip bipolar-current needle-knife (BB-knife) was developed for further easy use (6, 7). The electric current of the two bipolar knives is limited to the needle, leading to a reduction of perforation (8). Unfortunately, ESD procedure time is much longer by using a bipolar knife compared with a monopolar knife, mainly due to the lower cutting speed and cutting efficiency of a traditional bipolar knife (9).

**Figure 1:**
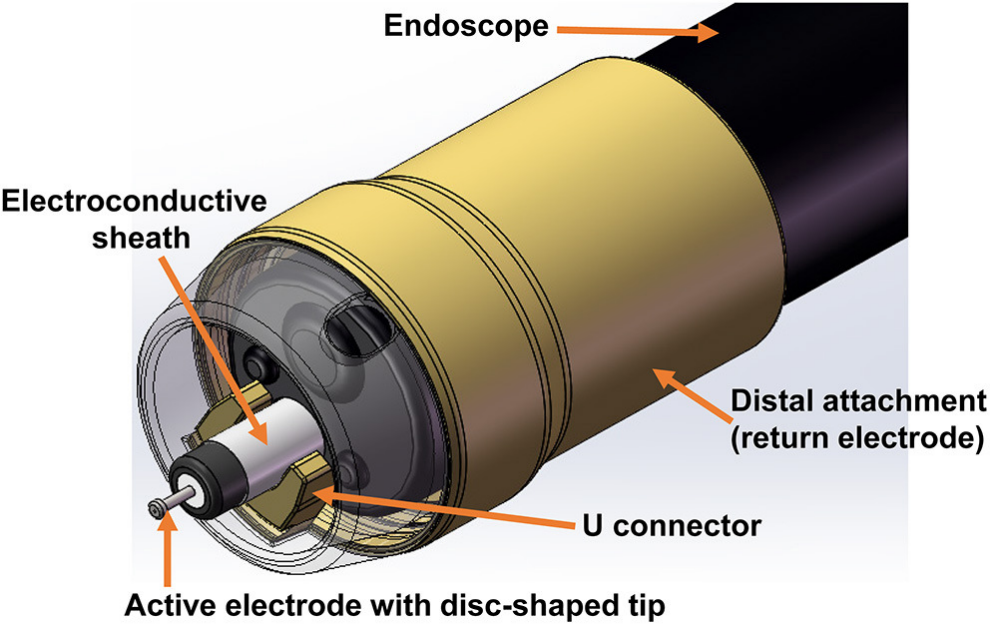
The structure of the novel bipolar knife.

Therefore, we developed a novel bipolar knife aimed to improve cutting speed while making sure of its safety during the ESD procedure. The main innovation of this bipolar knife was that the return electrode was assembled on a distal attachment outside of the endoscope rather than on the end of knife sheath (Figure 1), and the distal attachment can come into contact with the mucosa or mucus of the digestive tract and can conduct electricity during the ESD procedure. Given this structure, electric current can flow from the disc-shaped tip (active electrode) of the knife through a part of digestive tract superficial mucosa or mucus to distal attachment (return electrode), increasing the contact area between return electrode and tissue, which should theoretically improve cutting efficiency under the same voltage. Thus, we evaluated the cutting efficiency and feasibility of the novel bipolar knife for ESD in this study.

## Methods

### Animal Experiment

A total of 26 healthy pigs, no limitation with sex, aged 1–3 months, and weighted 30–40 kg, were purchased from Tianjin Bainong Laboratory Animal Breeding Technology Co. Ltd. We performed ESD for 15 mm target lesions in esophagus, stomach, and colorectum in 26 live pigs under general anesthesia. Acute and chronic time groups were divided in this study, and then animals were randomly assigned to monopolar and bipolar subgroups (Supplementary Table 1). In the acute time group, ESD was performed in 7 pigs by using monopolar knife and 7 pigs by using bipolar knife. These 14 pigs were sacrificed, and the specimens from esophagus, stomach and colorectum were taken immediately when the ESD procedure was completed. The remaining 12 pigs were belonged to the chronic time group, 6 of them were tested by using monopolar knife, and the other 6 were tested by using bipolar knife. After surgery, these 12 pigs were resuscitated, fed, and observed. On the 14th day, they were reexamined by an endoscope, euthanized, and autopsied, and specimens were taken by steps. Specifically, wound size, cutting time, en bloc resection rate, perforation, bleeding, thermal damage, and histopathological changes were recorded.

### Patients

**Figure 2:**
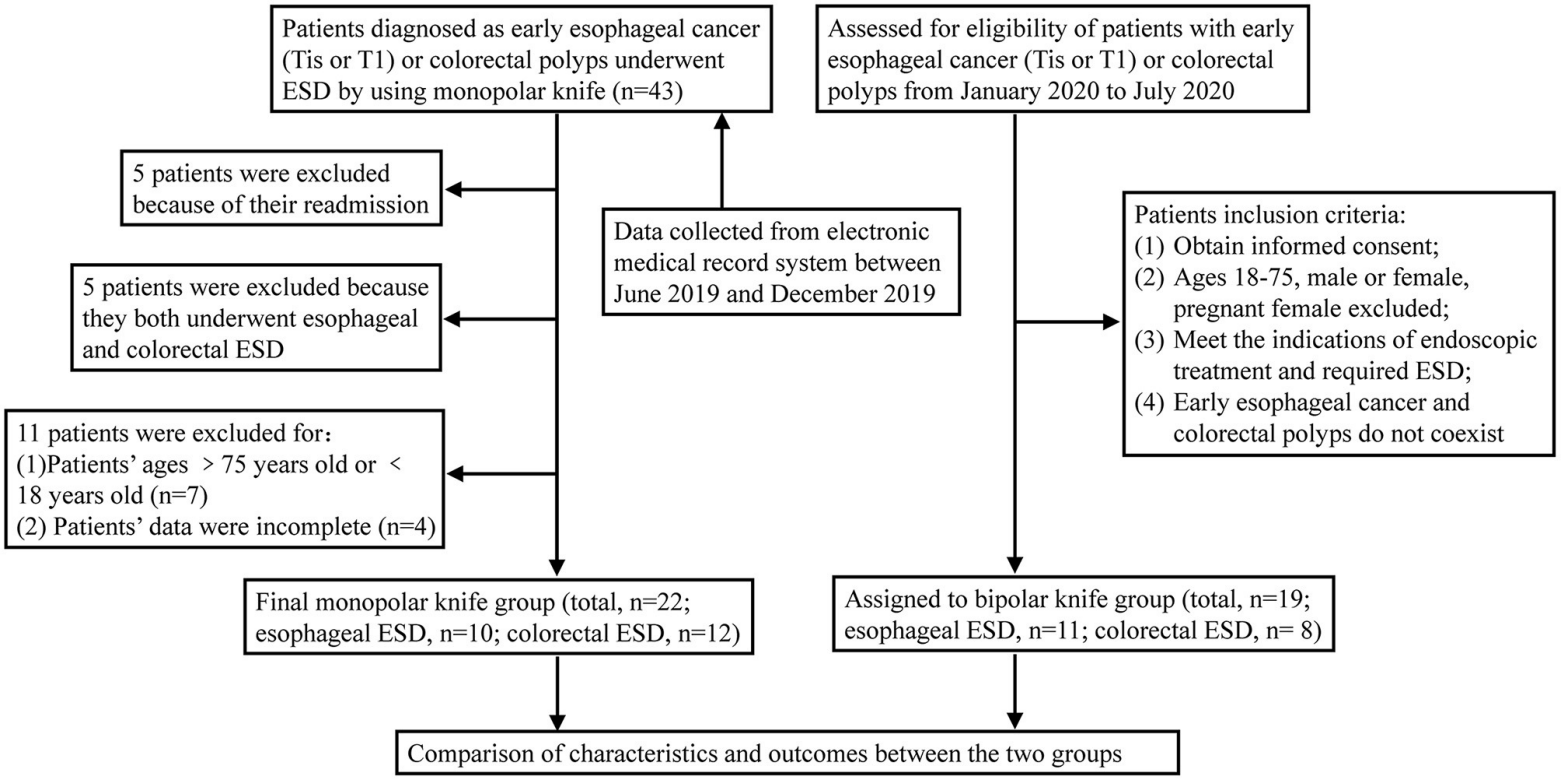
Flow diagram depicting the patient selection process.

A total of 19 patients were enrolled in the bipolar knife group (11 cases underwent esophageal ESD and 8 underwent colorectal ESD) according to the inclusion criteria. Data of 22 patients in the monopolar knife group were collected from the electronic medical record system between June 2019 and December 2019 (10 cases underwent esophageal ESD and 12 underwent colorectal ESD). All patients met the indications of ESD and provided their informed consent for the procedure. The detailed study design and inclusion criteria are summarized in Figure 2.

### Endoscopic Submucosal Dissection

We cut lesions using the monopolar and novel bipolar knives under an endoscope. The monopolar and bipolar knives used the same setting. The auto-cut mode was set at 30 W, and the forced coagulation mode was set at 60 W. ESD in our study was characterized by the following steps: (1) making markers on the mucosa surrounding the lesion; (2) injecting 0.9% saline and methylene blue mixture into the submucosa to gain a liquid pad with a diameter of about 1.5 cm and elevate the lesion; and (3) cutting the mucosa around the liquid pad and dissecting the submucosa from the edge of the lesion. Three endoscopists performed the ESD procedure. Before this study, they all had rich experience in ESD procedures and completed more than 1,000 ESD cases (Supplementary Table 2).

### Thermodynamic Damage Model

A thermodynamic damage model was developed to simulate an isolated digestive tract tissue in monopolar and bipolar systems by using the software COMSOL Multiphysics (version 5.4, COMSOL Inc.). The material and size of the electrodes were the same between monopolar and bipolar knives (Supplementary Figure 1). In the monopolar model, a ground electrode plate was attached to the bottom of the tissue, and the current flowed from the active electrode to the ground plate, whereas in the bipolar model, the current flowed from the active electrode to the return electrode that is assembled on the end of the endoscope (Supplementary Figure 2A).

For the purpose of further analysis of thermodynamic damage, the tissue of this model was simplified as a cuboid, and the active electrodes of the two knives were set to vertically insert tissue at 1 mm. We assumed that the heat flux and electric potentials of all boundaries met continuity. The active electrode was located in the center of the cuboid upper surface, and its thermal properties were the same as the surrounding area. The ground plate of the monopolar system was attached to the bottom of the tissue, and the return electrode of the bipolar system was simplified as a rectangular metal that positioned at the upper surface of the tissue (Supplementary Figure 2B).

### Statistical Analysis

To compare the results of animal experiment and patients' study between the monopolar and bipolar groups, the Mann–Whitney *U* test and chi-square test were used for continuous variables and dichotomous variables, respectively. All statistical analyses were performed using SPSS version 22.0 and GraphPad Prism version 8.0. The *P*-value was two-sided, and *P* < 0.05 indicated a statistical difference.

## Results

### Live Porcine Experiment

**Figure 3:**
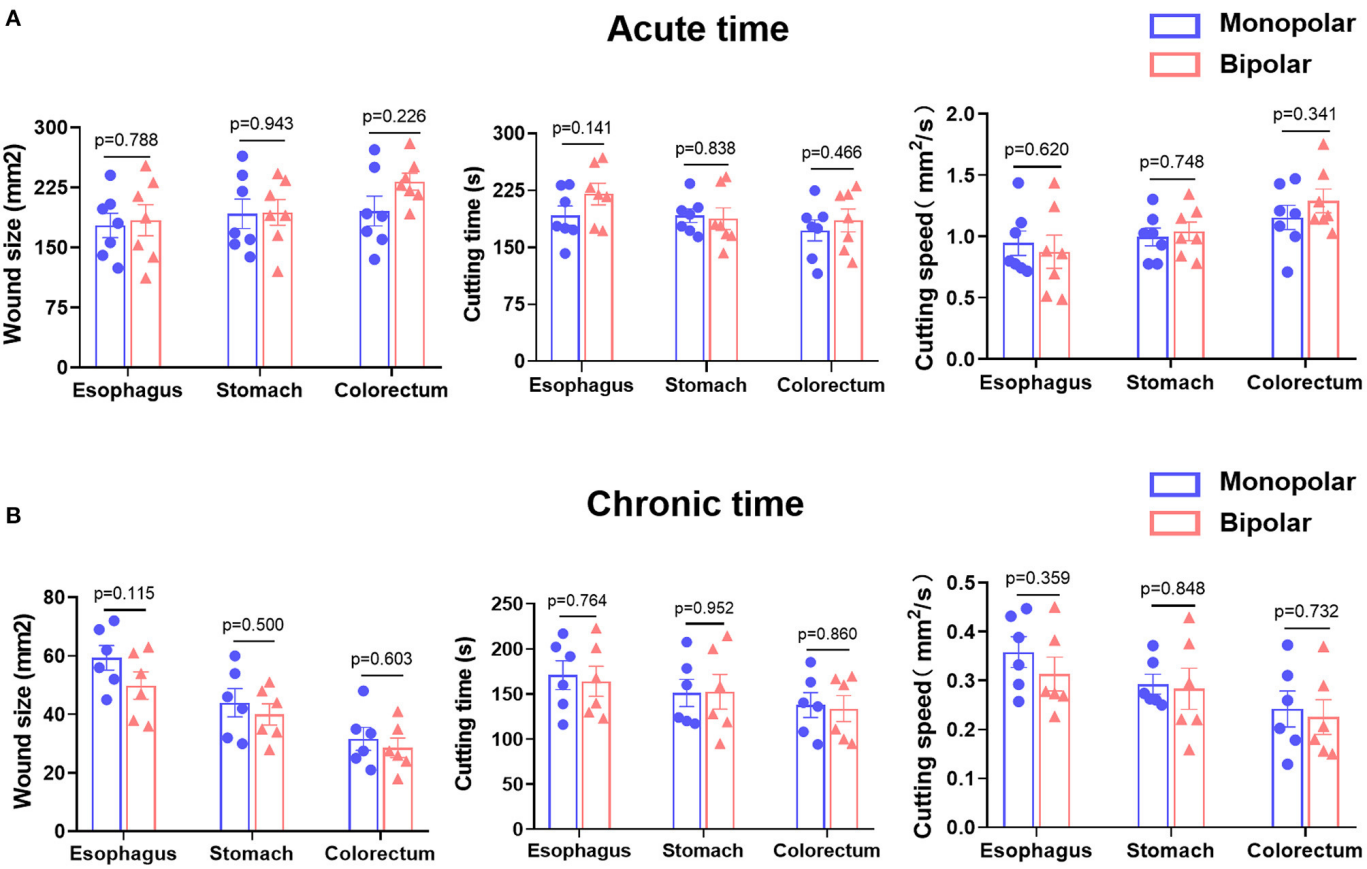
Comparison of wound size, cutting time, and cutting speed produced by monopolar and bipolar knives in porcine esophagus, stomach, and colorectum in the acute time group **(A)** and chronic time group **(B)**. Acute time group: record results immediately after endoscopic submucosal dissection (ESD). Chronic time group: record results on the 14^th^ day after ESD.

Both in the acute and chronic time groups, ESD procedure performed by the monopolar and novel bipolar knives showed no significant differences in wound size, cutting time, and cutting speed in esophagus, stomach, and colorectum (Figure 3). In the acute time group, the visible wounds of the digestive tract produced by the monopolar and bipolar knives looked similar; however, in the chronic time group, the digestive tract wounds caused by the two knives were almost healed (Supplementary Figure 3). The rates of en bloc resection in the monopolar and bipolar groups were both 100%. The rates of immediate and delayed bleeding between the monopolar and bipolar groups were also not statistically different. In addition, no perforation happened in the two groups during the animal experiments (Table 1).

**Table 1:** Ratio of en bloc resection, bleeding, and perforation in porcine experiment.

**Parameter**	**Monopolar group**		**Bipolar group**		***P***
	**(Event no./cutting no.)**	**%**	**(Event no./cutting no.)**	**%**	
En bloc resection	39/39	100	39/39	100	NA
Immediate bleeding	3/39	7.69	1/39	2.56	0.305
Delayed bleeding	2/18	11.1	1/18	5.60	0.546
Perforation	0/39	0	0/39	0	NA

*NA, not available*.

**Figure 4:**
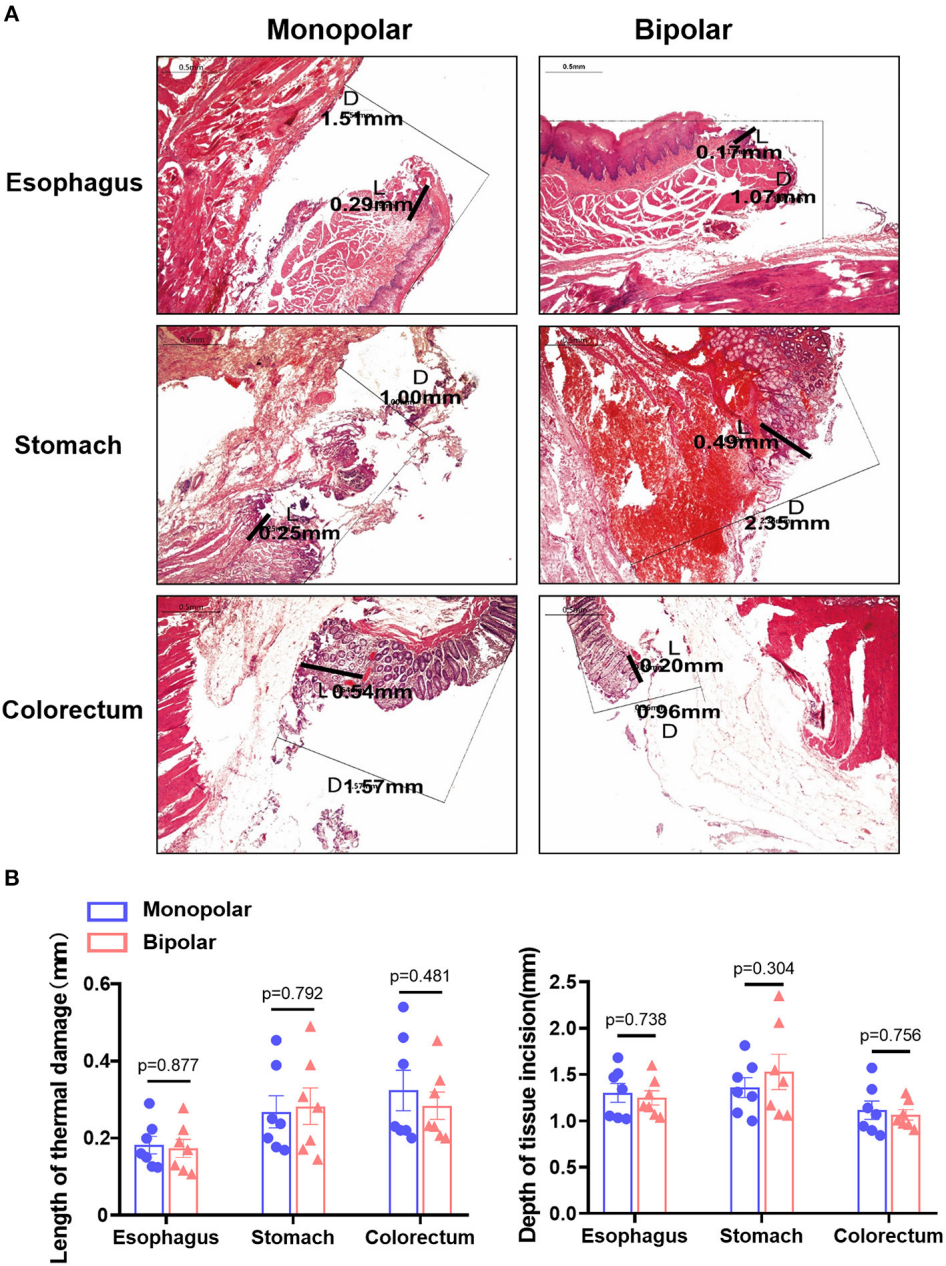
The digestive tract acute damage caused by the monopolar and bipolar knives during ESD operation. **(A)** H&E staining of porcine esophageal, gastric, and colorectal mucosa resected with the two knives. Scale bar: 0.5 mm. L: thermal damage length (range); D: incision depth; **(B)** Compare the length and depth of thermal damage between the monopolar and bipolar groups in esophagus, stomach, and colorectum at acute time point.

**Figure 5:**
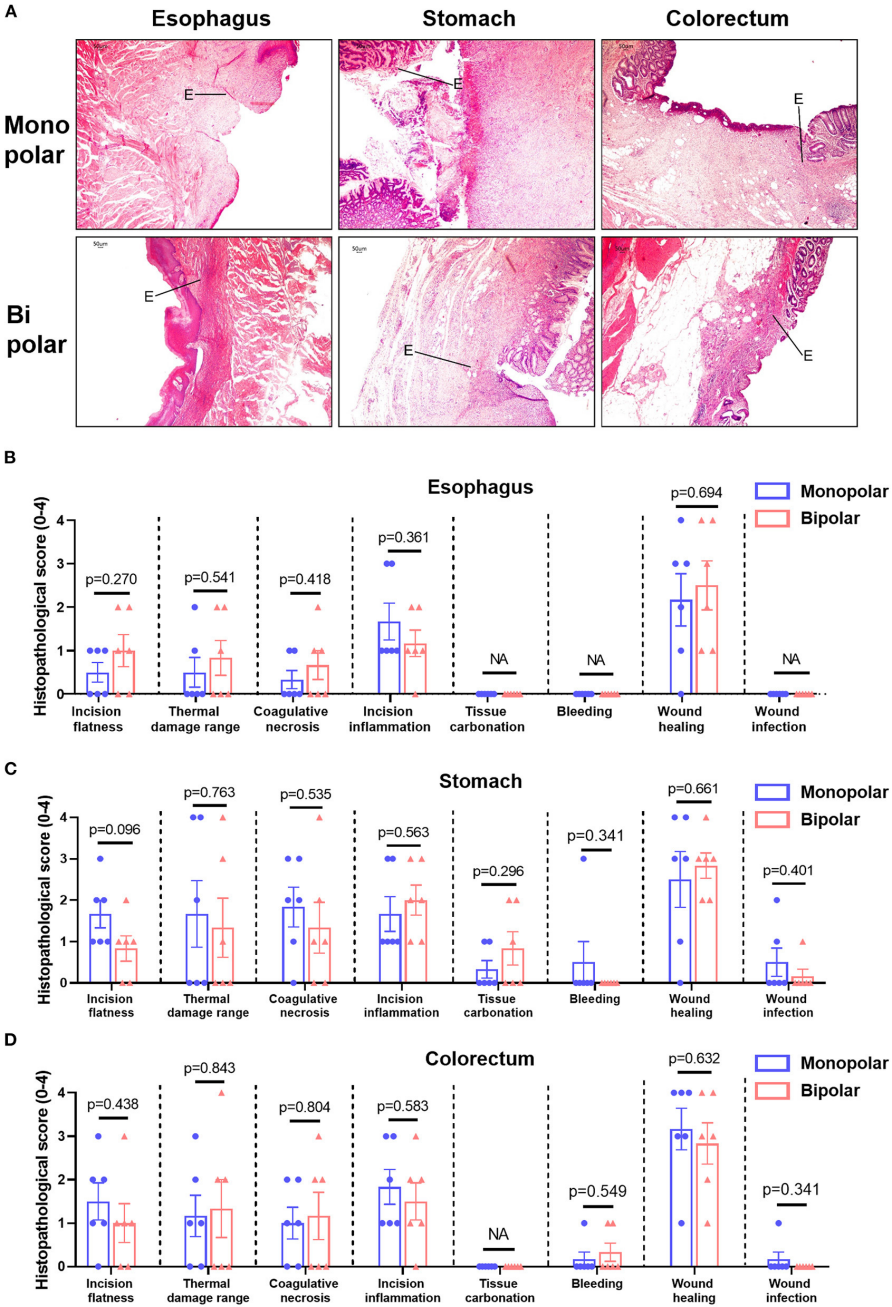
Histological changes of the digestive tract caused by the monopolar and bipolar knives in the chronic group. **(A)** Results of H&E staining about porcine esophageal, gastric and colorectal tissues resected by the two knives. Scale bar: 50 μm. The edges of wounds were indicated by solid line. Comparison of histological scores on incision flatness, thermal damage range, coagulative necrosis, incision inflammation, tissue carbonation, bleeding, wound healing, and wound infection between monopolar and bipolar groups in esophagus **(B)**, stomach **(C)**, and colorectum **(D)**. The evaluation standards of these histological scores are summarized in Supplementary Table 3.

Histological changes in the acute time group were assessed in terms of thermal damage length and incision depth, while histological changes in the chronic time group were presented as histopathological scores. The histological changes of target lesions in esophagus, stomach, and colorectum produced by monopolar and bipolar knives in the acute time group are shown in Figure 4A, and the length and depth of thermal damage caused by these two knives did not show any significant difference (Figure 4B). For the chronic time group, the histological changes caused by the two knives are shown in Figure 5A. In esophagus, stomach, and colorectum, the histopathological scores between monopolar and bipolar subgroups were not significantly different on incision flatness, thermal damage range, coagulative necrosis, incision inflammation, tissue carbonation, bleeding, wound healing, and wound infection (Figures 5B–D).

### ESD in Patients

In the patients' study, a total of 21 esophageal ESDs and 20 colorectal ESDs were performed. For esophageal ESD, 10 and 11 patients were assigned to the monopolar group and bipolar group, respectively. For colorectal ESD, 12 and 8 patients were enrolled into the monopolar knife group and bipolar knife group, respectively. The baseline clinical characteristics, such as age, sex, smoking, alcohol drinking, family cancer history, hypertension, and diabetes mellitus, were almost the same between the monopolar and bipolar groups (Table 2). As shown in Tables 3, 4, the characteristics of target lesions in esophagus and colorectum were not significantly different between the groups of two knives. Regarding histological classification, squamous carcinoma was the most common type in esophageal lesions in these two groups (60 vs. 63.6%); for colorectal polyps, the high-grade adenoma and hyperplastic polyp were the most common type, respectively, in the two groups.

**Table 2:** The characteristics of patients underwent ESD in monopolar and bipolar groups.

**Parameter**	**Esophageal ESD (*****n*** **=** **21)**			**Colorectal ESD (*****n*** **=** **20)**		
	**Monopolar (*n* = 10)**	**Bipolar (*n* = 11)**	***P***	**Monopolar (*n* = 12)**	**Bipolar (*n* = 8)**	***P***
Age (years), mean ± SD	62.80 ± 8.95	67.36 ± 7.58	0.221	54.17 ± 11.07	56.88 ± 12.19	0.613
Male sex, *n* (%)	7 (70.0)	10 (90.9)	0.311	5 (41.7)	3 (37.5)	1.000
Smoking, *n* (%)	6 (60.0)	6 (54.5)	1.000	1 (8.3)	1 (12.5)	1.000
Alcohol drinking, *n* (%)	4 (40.0)	7 (63.6)	0.395	3 (25.0)	0 (0)	0.242
Family cancer history, n (%)	4 (40.0)	6 (54.5)	0.670	3 (25.0)	6 (75.0)	0.065
Hypertension, *n* (%)	4 (40.4)	6 (54.5)	0.670	4 (33.3)	2 (25.0)	1.000
Diabetes mellitus, *n* (%)	1 (10.0)	1 (9.1)	1.000	1 (8.3)	2 (25.0)	0.537
Preoperative CRP (mg/L), mean ± SD	2.09 ± 2.19	1.35 ± 1.68	0.392	1.08 ± 0.79	1.18 ± 0.91	0.786
Preoperative WBC count (109/L), mean ± SD	5.26 ± 1.03	4.85 ± 1.17	0.402	6.56 ± 2.19	6.19 ± 2.44	0.727
Preoperative neutrophil count (109/L), mean ± SD	3.30 ± 0.76	2.93 ± 0.92	0.327	4.11 ± 1.58	3.64 ± 1.56	0.519
Preoperative neutrophil percentage (%), mean± SD	62.59 ± 6.95	59.21 ± 8.26	0.326	61.99 ± 7.54	57.86 ± 7.10	0.235

*SD, standard deviation; CRP, C-reactive protein; WBC, white blood cell. P: dichotomous variables—Chi-square test or Fisher's exact test; continuous variables—Mann–Whitney U test*.

**Table 3:** Characteristics of esophageal lesions in patients who underwent esophageal ESD.

**Parameter**	**Monopolar (*n* = 10)**	**Bipolar (*n* = 11)**	***P***
Lesion size (cm^2^), mean ± SD	13.83 ± 11.53	7.84 ± 3.81	0.119
Position, *n* (%)			0.365
Cervical esophagus	1 (10.0)	0 (0)	
Upper thoracic esophagus	2 (20.0)	1 (9.1)	
Middle thoracic esophagus	5 (50.0)	4 (36.4)	
Lower thoracic esophagus	2(20.0)	6 (54.5)	
IPCL classification, *n* (%)			1.000
B1	9 (90.0)	10 (90.9)	
B2	1 (10.0)	1 (9.1)	
Histopathology, *n* (%)			0.459
Low-grade intraepithelial neoplasia	1 (10.0)	0 (0)	
High-grade intraepithelial neoplasia	3 (30.0)	4 (36.4)	
Squamous carcinoma	6 (60.0)	7 (63.6)	

*SD, standard deviation. IPCL classification: intraepithelial papillary capillary loop classification. P: dichotomous variables—Chi-square test or Fisher's exact test; continuous variables—Mann–Whitney U test*.

**Table 4:** Characteristics of colorectal polyps in patients who underwent colorectal ESD.

**Parameter**	**Monopolar (*n* = 12)**	**Bipolar (*n* = 8)**	***P***
Polyp maximum size (cm), mean ± SD	1.69 ± 1.47	1.34 ± 0.47	0.521
Morphology, *n* (%)			0.642
Granular	7 (58.3)	6 (75.5)	
Non-granular	5 (41.7)	2 (25.0)	
Pit pattern classification, *n* (%)			0.450
I	3 (25.0)	2 (25.0)	
II	1 (8.3)	3 (37.5)	
III-L	3 (25.0)	1 (12.5)	
III-S	0 (0)	0 (0)	
IV	3 (25.0)	0 (0)	
V	2 (16.7)	2 (25.0)	
Histopathology, *n* (%)			0.621
Inflammatory polyp	2 (16.7)	0 (0)	
Hyperplastic polyp	1 (8.3)	3 (37.5)	
Adenoma	2 (16.7)	1 (12.5)	
High-grade adenoma	4 (33.3)	1 (12.5)	
Adenocarcinoma	1 (8.3)	1 (12.5)	
Carcinoid	2 (16.7)	2 (25.0)	

*SD, standard deviation. P: dichotomous variables—Chi-square test or Fisher's exact test; continuous variables—Mann–Whitney U test*.

The outcomes and adverse events of ESDs in the two groups are presented in Table 5. All lesions of both groups achieved en bloc resection. No immediate bleeding, delayed bleeding, or perforation happened in the monopolar and bipolar groups. In esophageal ESD, 2 patients in the bipolar group had infection, but did not show a statistical difference compared with the monopolar group. Infection was not found in both groups after colorectal ESD. In addition, the postoperative inflammatory markers, such as C-reactive protein, white blood cell count, neutrophil count, and neutrophil percentage, were not significantly different between monopolar and bipolar groups.

**Table 5:** Outcomes and adverse events of patients after ESD in monopolar and bipolar groups.

	**Esophageal ESD (*****n*** **=** **21)**			**Colorectal ESD (*****n*** **=** **20)**		
**Parameter**	**Monopolar (*n* = 10)**	**Bipolar (*n* = 11)**	***P***	**Monopolar (*n* = 12)**	**Bipolar (*n* = 8)**	***P***
En bloc resection, *n* (%)	10 (100)	11 (100)	NA	12 (100)	8 (100)	NA
Adverse events, *n* (%)						
Immediate bleeding^*^	0 (0)	0 (0)	NA	0 (0)	0 (0)	NA
Delayed bleeding^*^	0 (0)	0 (0)	NA	0 (0)	0 (0)	NA
Perforation	0 (0)	0 (0)	NA	0 (0)	0 (0)	NA
Infection	0 (0)	2 (18.2)	0.476	0 (0)	0 (0)	NA
Postoperative CRP (mg/L), mean ± SD	34.9 ± 15.94	31.09 ± 17.59	0.606	7.74 ± 10.79	9.48 ± 13.20	0.749
Postoperative WBC count (10^9^ /L), mean ± SD	6.84 ± 1.00	6.98 ± 1.63	0.815	5.53 ± 1.42	5.25 ± 1.81	0.708
Postoperative neutrophil count (10^9^ /L), mean ± SD	5.03 ± 0.83	5.27 ± 1.64	0.678	3.49 ± 1.65	3.34 ± 1.28	0.783
Postoperative neutrophil percentage (%), mean ± SD	73.72 ± 6.19	74.70 ± 5.63	0.708	62.69 ± 7.00	62.33 ± 6.33	0.907
Length of in-hospital stay (days), mean ± SD	4.80 ± 1.40	4.00 ± 1.00	0.145	4.25 ± 1.71	3.63 ± 0.92	0.359

*SD, standard deviation; CRP, C-reactive protein; WBC, white blood cell; NA, not available*.

* *Immediate bleeding defined as hemorrhage during the procedure*.

* *Delayed bleeding defined as bleeding that occurred at least 1 h after the procedure. P: dichotomous variables—Chi-square test or Fisher's exact test; continuous variables—Mann–Whitney U test*.

### Finite Element Analysis of Monopolar and Bipolar Knives

**Figure 6:**
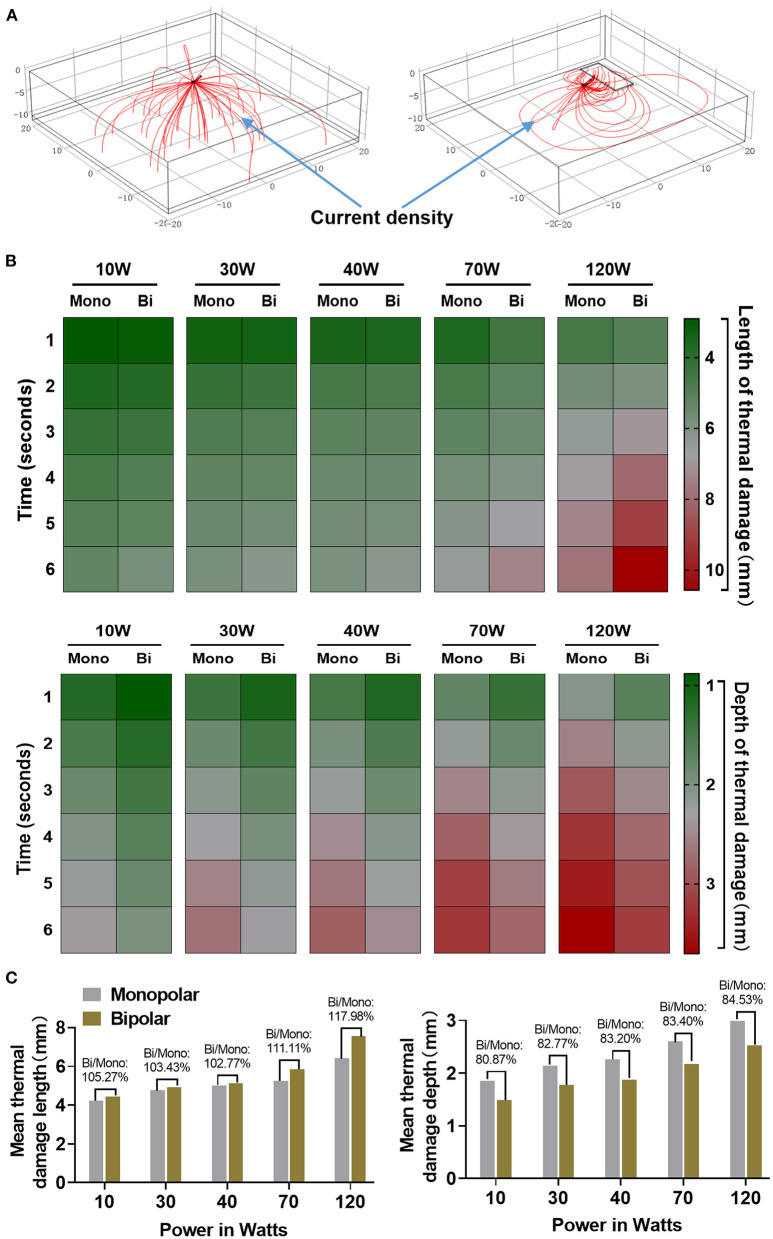
The finite element analysis of thermal damage caused by the monopolar and bipolar knives. **(A)** Current density streamline diagrams of monopolar and bipolar electrosurgical models. **(B)** The horizontal and vertical thermal damage analyzed by the finite element method under power of 10, 30, 40, 70, and 120 W with different times between monopolar and bipolar knives. **(C)** The bipolar-to-monopolar ratio of the average thermal damage length and depth under different powers between two knives.

The current density streamlines of the two knives based on a short-term transient finite element analysis of 1 s are shown in Figure 6A. The current of the monopolar knife model passed through the full-layer tissue from the knife tip vertically downward. The current density of the central region was the largest, which gradually decreased outward. In the bipolar knife model, the current flowed from the knife tip to the return electrode, and the current density of the upper surface was the largest, which gradually decreased downward. Besides, we compared the length and depth of thermal damage (horizontal and vertical damage) caused by the two knives in the region with temperature over 43°C at a different time under the power of 10, 30, 40, 70, and 120 W (Figure 6B). The average lengths of thermal damage in the monopolar model were 4.22, 4.77, 5.00, 5.26, and 6.41 mm under the power above, and those in the bipolar model were 4.45, 4.93, 5.14, 5.85, and 7.57 mm. At the same power condition, the depths of thermal damage produced by the monopolar knife were 1.85, 2.14, 2.26, 2.60, and 2.99 mm, while those produced by the bipolar knife were 1.49, 1.77, 1.88, 2.17, and 2.53 mm. Thus, the bipolar-to-monopolar percentages of average length and depth of thermal damage were, respectively, 102.77–117.98% and 80.87–84.53% in the finite element model (Figure 6C).

## Discussion

The ESD has the advantages of a high en bloc resection rate and a low local recurrence rate, and yields an accurate pathological diagnosis, although substantial procedural complications (e.g., perforation) have been reported (1, 10). Following the introduction of an insulation-tipped diathermic knife (IT knife), many other electrosurgical knives have been reported for ESD (5, 11). Electrosurgical knives can be divided into monopolar knives and bipolar knives according to different current circuits. A monopolar knife has one electrode and one grounding pad, which is attached to the body surface. The current flow of the monopolar knife passes through the human body from the active electrode to the generator *via* the grounding pad (8). The vertical current transmission can cause thermal injury to the deep tissue of the digestive tract, leading to the occurrence of complications associated with ESD, such as perforation (4).

In contrast, a bipolar knife has two electrodes, and the current flow of the bipolar knife passes through the tissue horizontally from the active electrode to the generator *via* the return electrode. The current is horizontally transmitted and only limited between the two electrodes (8). Thus, the vertical thermal damage to the tissue caused by the bipolar knife is reduced within the controllable range of the surgical field, which may minimize the frequency and severity of complications associated with ESD (4). In addition, compared with the monopolar knife, the circuit feature of bipolar knife may make itself more friendly for patients who have medical devices' implantation. Because the current flow of bipolar knife only passes through a small area between the two electrodes, its electrical signal rarely interferes the implanted medical devices, such as artificial pacemaker and defibrillator (12).

This study evaluated the safety and feasibility of a novel bipolar needle knife for ESD in animals and patients. The perforation was not found in the monopolar group and bipolar group, and the en bloc resection of both groups was totally completed. In addition, the incidence of immediate and delayed bleeding did not show any significant difference between the monopolar and bipolar groups in pigs and patients (Tables 1, 5). The wound size, length of thermal damage, and depth of thermal damage were almost the same in monopolar and bipolar groups (Figures 3, 4). In the chronic time group, histopathological results of the porcine experiment showed that incision flatness, thermal damage range, coagulative necrosis, incision inflammation, tissue carbonation, bleeding, wound healing, and wound infection all were not significantly different between the monopolar and the bipolar subgroups in esophagus, stomach, and colorectum (Figure 5). Hence, we confirmed that the safety of the bipolar knife was at least not inferior to the monopolar knife for ESD according to the results of the porcine and patients' study.

To date, ESD knives are still dominated by monopolar knives clinically because of the low cutting speed of traditional bipolar knives. The most important point of this study is that our novel bipolar knife was proved that it has almost the same cutting speed as that of a commonly used monopolar knife in porcine ESD (Figure 3), meaning that the cutting efficiency of our bipolar knife is superior to the previously invented bipolar knives (i.e., the BB-knife). During the actual ESD operation with traditional bipolar knife, return electrode needs to be placed on the wall of the lumen all the time and contacts the gastrointestinal mucosa to form a current circuit. Therefore, once ESD is performed in a large lumen, such as gastric angulus and body, the return electrode will be suspended and difficult to touch the gastrointestinal mucosa, which hinders the current circuit formation. However, the innovation of our novel bipolar knife is that the return electrode is assembled on a distal attachment at the end of the endoscope but not on the end of the knife sheath (Figure 1). The current can flow from the tip of the knife to the distal attachment through mucosa or mucus of the digestive tract surface, and then back to the high-frequency generator *via* the return electrode, making full use of the peripheral surface area of the endoscope. In addition, the end part of the knife sheath is also electroconductive, and a small current circuit can be formed when it touched the mucosa or mucus on the gastrointestinal surface, which makes sure that the novel bipolar knife can still work when the contact area with the mucosa of digestive tract is limited. The active electrode, the end of knife sheath, and the distal attachment of this novel bipolar knife are all electroconductive. This special characteristic increases the conductive area between tissue and knife, makes the current circuit easier to form than the traditional bipolar knife, enhances the current intensity of knife tip, and finally improves cutting efficiency under the same voltage.

What calls for special attention is that the knife tip and the end of the endoscope need to keep a certain distance during the ESD procedure by using this bipolar knife, and the knife tip should be clearly seen in the surgical field of vision. If the knife tip is too far from the end of the endoscope, it is difficult for the distal attachment to touch the mucosa, resulting in the contact area between the return electrode and the mucosal tissue becomes smaller, the current intensity of the knife tip is reduced, and at last the cutting efficiency is lowered. By the way, the tip of this novel bipolar knife was shaped as a disc (Figure 1), which provides an anti-slip effect, and makes it easier to mark, hook, and cut the tissue during ESD operation.

The peristaltic frequency of the digestive tract and thickness of its mucosa were different among porcine and human participants. Besides, the real digestive tract structure is complex and layered, and its electrical resistance is uneven. Although endoscopists in this study all have rich operating experience in ESD, their operating skills still cannot be totally consistent. For these reasons, the results of this study may have some deviations. To overcome the deviations caused by these confounding factors above, we then evaluated the safety and effectiveness of the bipolar knife by using the finite element method. The structure of the simulated tissue in the finite element model was simple, and electrical resistance in different parts of the simulated tissue was consistent. In addition, the finite element analysis can well-simulate the specific potential distribution and current density. The simulated model showed that the current flow of the monopolar system was vertical and the current density gradually decreased outward, whereas the current flow of the bipolar device was horizontal and its current density gradually decreased downward (Figure 6A). Moreover, the mean length and depth of thermal damage produced by the bipolar knife were, respectively, 102.77–117.98% and 80.87–84.53% of those produced by the monopolar knife at the same power (Figure 6C). Therefore, the bipolar knife might be safer than its monopolar counterpart for ESD procedure due to the theoretical reduction of thermal damage depth.

In summary, this study demonstrated that our novel bipolar needle knife had similar cutting efficiency to the monopolar knife, and the safety was at least not inferior to its monopolar counterpart. Furthermore, the finite element analysis showed that this bipolar knife may tend to be safer than the monopolar knife. Thus, we concluded that our novel bipolar needle knife can be an alternative device choice for ESD based on that it not only ensures the cutting efficiency but also theoretically reduces the electrical damage during the cutting process. It is a pity that this was not a truly prospective study. The data of the monopolar group were retrospectively collected from the electronic medical record system and might have a selection bias. Moreover, the sample size of our study was small, and all of the participants were only recruited from one institute. Therefore, a larger sample size prospective study with balanced populations from multiple centers is required to further validate the safety and feasibility of our novel bipolar knife in the future.

## Data Availability Statement

The raw data supporting the conclusions of this article will be made available by the authors, without undue reservation.

## Ethics Statement

The studies involving human participants were reviewed and approved by Ethics Committee of Zhejiang Cancer Hospital. The patients/participants provided their written informed consent to participate in this study. The animal study was reviewed and approved by Ethics Committee of Zhejiang Cancer Hospital.

## Author Contributions

SW conceived the idea and designed experiments. DZ, YuL, YaL, and QS collected data. SC and DZ analyzed the data and drafted the manuscript. SW, JY, and RR performed the endoscopic resection. All authors contributed to the article and approved the submitted version.

## Conflict of Interest

The authors declare that the research was conducted in the absence of any commercial or financial relationships that could be construed as a potential conflict of interest.

## Publisher's Note

All claims expressed in this article are solely those of the authors and do not necessarily represent those of their affiliated organizations, or those of the publisher, the editors and the reviewers. Any product that may be evaluated in this article, or claim that may be made by its manufacturer, is not guaranteed or endorsed by the publisher.

## Abbreviations

ESD: Endoscopic submucosal dissection
SD: Standard deviations
CRP: C-reactive protein
WBC: White blood cell.
