# Supplementary material for: A Novelly Developed Bipolar Needle Knife Can Be an Alternative Device Choice for Endoscopic Submucosal Dissection (With Video)
Source: Front Med (Lausanne). 2022 May 13;9:888635. doi: 10.3389/fmed.2022.888635 (PMC9136242; doi:10.3389/fmed.2022.888635)
Supplement: Supplementary file 1 [file Data_Sheet_1.docx]

**A novelly developed bipolar needle knife can be an alternative device choice for endoscopic submucosal dissection (with video)**

Shengsen Chen^†^, MD, Danping Zhou^†^, MD, Jiangping Yu^†^, MD, Rongwei Ruan^†^, MD, Yuanshun Liu, MD, Yandong Li, MD, Qiwen Shen, MD, Shi Wang^*^, MD

Department of Endoscopy, Cancer Hospital of the University of Chinese Academy of Sciences (Zhejiang Cancer Hospital), Institute of Basic Medicine and Cancer (IBMC), Chinese Academy of Sciences, Hangzhou, China.

^*^Corresponding author: Shi Wang, e-mail: wangshi@zjcc.org.cn, telephone number: (+86) +86-571-88122277, fax number: (+86) +86-571-88122277. Complete correspondence address: Zhejiang Cancer Hospital, No. 1 Banshandong Road, Hangzhou, 310022

Funding：None.

^†^These authors are contributed equally to this work.

**Supplementary** **Figure 1. Structure comparison of monopolar and bipolar knife**

**Supplementary** **Figure 2. ESD knife test model established by finite element method.** (A) Electric cutting experimental model of monopolar and bipolar knives. Monopolar model: referring to the clinical use, the inclination angle is 60°, the ground electrode plate is attached to the lower surface of the tissue, and the current forms a circuit from the active electrode through the tissue to the ground electrode plate. Bipolar model: referring to the clinical use, the inclination angle is 60°, the return electrode is attached to the upper surface of the tissue, and the current forms a circuit from the active electrode through the tissue to the another electrode.(B) Simplified model of electric cutting experiment for monopolar and bipolar knives.

**Supplementary** **Figure 3. Animal** **autopsy photograph after ESD.** Wounds of digestive tract (esophagus and colon) produced by monopolar and bipolar knives in acute time group and chronic time group. In chronic time group, the autopsy photograph showed the healed wounds of esophageal and colonic mucosas.


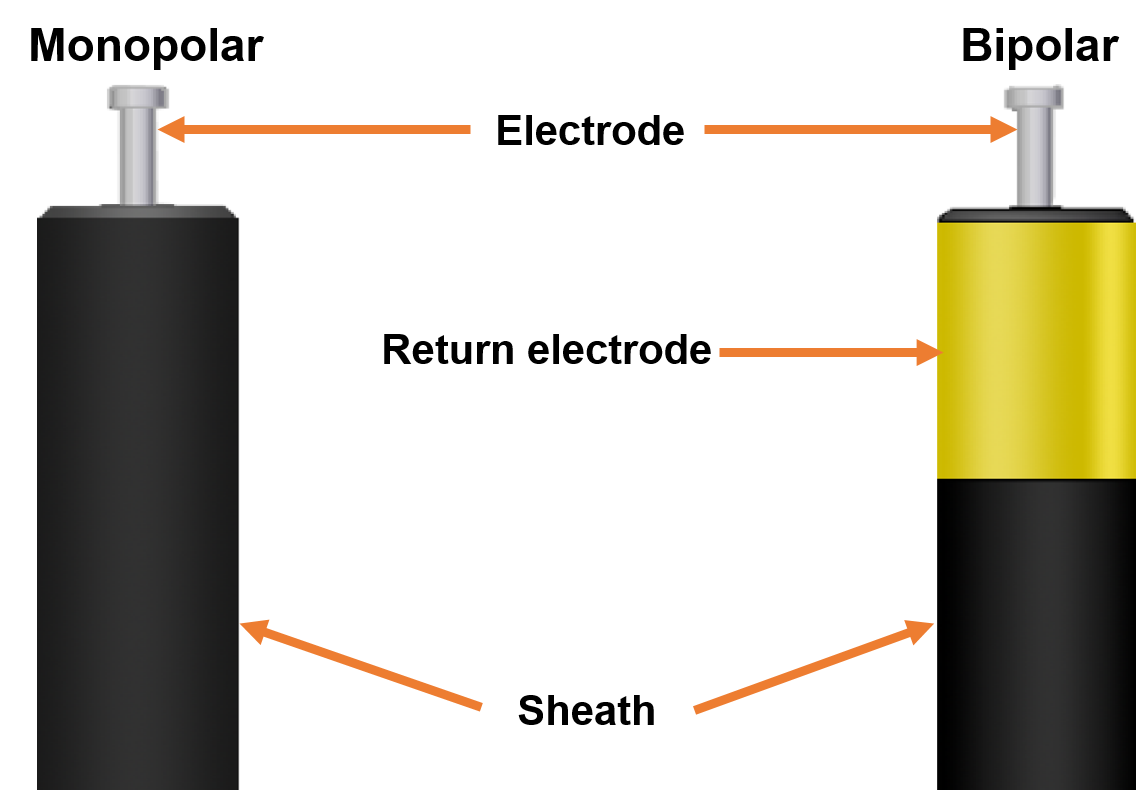


**Supplementary** **Figure 1**


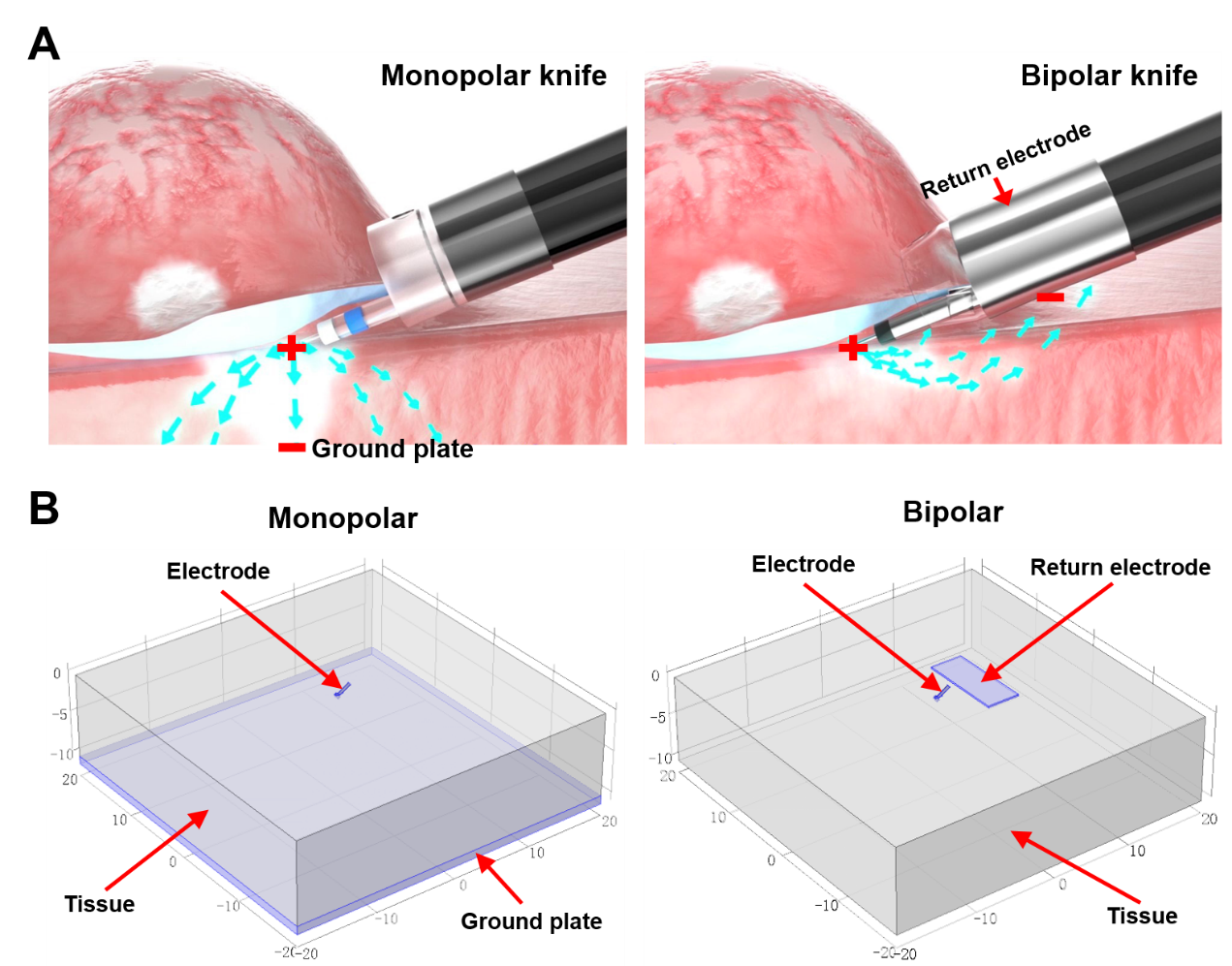


**Supplementary** **Figure 2**

**
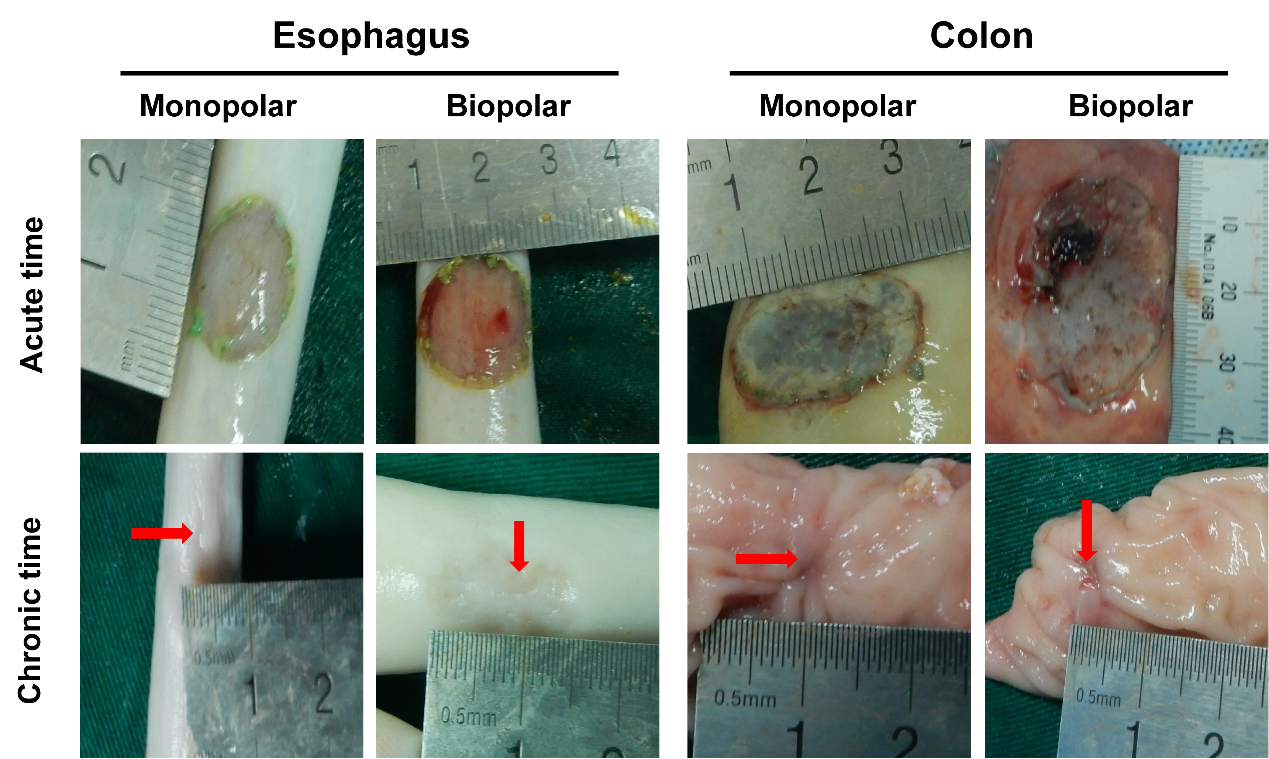
**

**Supplementary** **Figure 3**

| **Supplementary** **Table 1.** The group design of animal experiment. | | | | | |
| --- | --- | --- | --- | --- | --- |
| **Time of animal sacrificed** | **Group** | **Number of pigs** | **Number of ESD resections** | | |
|  |  |  | **Esophagus** | **Stomach** | **Colorectum** |
| Acute time | Monopolar | 7 | 7 | 7 | 7 |
|  | bipolar | 7 | 7 | 7 | 7 |
| Chronic time | Monopolar | 6 | 6 | 6 | 6 |
|  | bipolar | 6 | 6 | 6 | 6 |

| **Supplementary** **Table 2.** Characteristics of the 3 endoscopists. | | | |
| --- | --- | --- | --- |
| **Characteristic** | **Endoscopist 1** | **Endoscopist 2** | **Endoscopist 3** |
| Sex | Male | Male | Male |
| Age(years) | 42 | 45 | 47 |
| No. of endoscopies conducted, n | ＞10,000 | ＞10,000 | ＞10,000 |
| No. of cases for ESD, n | ＞1,000 | ＞1,000 | ＞1,000 |

| **Supplementary** **Table 3.** The histological evaluation standard. | | | | | |
| --- | --- | --- | --- | --- | --- |
| **Parameters** | **Pathological features and their corresponding scores** | | | | |
|  | **Score 0** | **Score 1** | **Score 2** | **Score 3** | **Score 4** |
| **Incision flatness** | Cutting surface flattening | Cutting surface is slightly uneven | Cutting surface is uneven | Cutting surface is extremely uneven | The incision surface lose the original structure |
| **Coagulative necrosis** | No coagulative necrosis around the incision | Slight coagulative necrosis around the incision | Moderate coagulative necrosis around the incision | Moderate to obvious coagulative necrosis around the incision | Obvious coagulative necrosis around the incision |
| **Incision inflammation** | No inflammatory cells around the incision | Small amount of inflammatory cell infiltration around the incision | Mild dense inflammatory cell infiltration around the incision | Moderate or above dense inflammatory cell infiltration around the incision | Patches of inflammatory cells infiltration around the incision |
| **Tissue carbonization** | No carbonization around the incision | Occasional carbonization around the incision | Small amount of carbonization around the incision | Many carbonization around the incision | Large carbonization area around the incision |
| **Bleeding** | No bleeding | Occasional bleeding around the tissue | Small but significant bleeding around the tissue | Significant large bleeding area around the tissue | Massive bleeding around the tissue |
| **Wound healing** | No tissue healing at the incision | The tissue at the incision edge is slightly hyperplastic | The tissue at the incision extends from the edge to the middle and is intended to heal | Moderate to significant healing of the tissue at the incision | The tissue at the incision is obviously healed |
| **Wound infection** | No infection, no bacterial colonization | Minor infection | Moderate infection | Moderate to severe infection | Severe infection |
